# Synergistic application of controlled-release urea and potassium chloride to enhance wheat yield and nutrient use efficiency

**DOI:** 10.3389/fpls.2026.1839500

**Published:** 2026-05-20

**Authors:** Xiuyi Yang, Jianbang Li, Wenxiao Zhang, Wenjun Jia, Zeli Li, Jibiao Geng, Shutong Lei, Hui Li, Qingping Zhang, Ying Lang, Xianqi Huo

**Affiliations:** College of Agriculture and Forestry Science, College of Resources and Environment, Linyi University, Linyi, Shandong, China

**Keywords:** controlled-release potassium chloride, controlled-release urea, nutrient use efficiency, wheat, yield

## Abstract

Nitrogen and potassium fertilization played a central role in promoting the growth of wheat. A three-year (2022-2024) split-plot field experiment was conducted to investigate the impacts of combining controlled-release urea (CRU) with controlled-release potassium chloride (CRK) on nutrient leaching and use efficiency in wheat fields. In this study, the control plot received neither nitrogen nor potassium applications (Control). The main plots were designated based on nitrogen fertilizer types, including controlled-release urea (CRU) and conventional urea (Urea). The sub-plots were assigned potassium fertilizer rates using CRK, specifically 50 kg ha-1 (LCRK), 75 kg ha-1 (MCRK), and 100 kg ha-1 (HCRK). The wheat yields in CRU treatments witnessed a modest average increase of 2.2% from 2022 to 2024 compared to ordinary urea treatments. In the final season, nitrogen recovery efficiency augmented by 10.9%. Furthermore, CRU treatments boosted the number of wheat spikes and grains per spike but had no notable influence on the thousand-grain weight (TGW) of wheat. Consequently, the yield enhancement observed in CRU treatments was primarily attributed to an increase in tiller count of wheat. The application of CRU×MCRK modestly improved wheat leaf photosynthesis during its mid to late growth stages, nutrient use efficiency and potential environmental benefits.

## Introduction

In modern times, wheat had become one of the most important food crops in the world, with a consistent rise in yield being the paramount approach to fulfilling market demands and safeguarding food security (Mottaleb et al., 2022). However, the productivity and quality of wheat were shaped not only by the genetic traits of the cultivar but also by the intricate interplay between the ecological environment and agricultural practices (Guarin et al., 2022). Among the various agricultural strategies employed, the application of mineral fertilizers proved to be the most rapid, effective, and crucial means of enhancing crop yields. Rational use of chemical fertilizers played a pivotal role in both ensuring food production safety and achieving high yields (Jiang et al., 2021).

Nitrogen was a vital nutrient in the growth trajectory and development of wheat, playing a pivotal role in fostering robust root systems and facilitating the accumulation of essential elements like carbon and potassium (de Oliveira Silva et al., 2020). When nitrogen fertilizer was judiciously applied, it boosted wheat chlorophyll levels, enhanced photosynthetic processes, augmented the accumulation of photosynthetically derived products, extended the functionality of green leaves, and ultimately led to higher wheat yields (Liu et al., 2024). In parallel, potassium emerged as another indispensable nutrient crucial for plant growth and development (Wang et al., 2020). The application of potassium fertilizer invigorated plant root systems, enhanced the uptake and accumulation of nitrogen and phosphorus, facilitated the efficient transportation and distribution of assimilates towards ear organs, amplified sucrose supply and starch accumulation within grains during the grain-filling phase, and ultimately elevated economic yields (Khan et al., 2022).

Nevertheless, achieving high yields often came at the expense of unreasonable fertilization practices. These practices not only resulted in the squandering of resources and a decline in fertilizer utilization efficiency but also triggered a cascade of adverse effects, including plant diseases, soil acidification, agricultural non-point source pollution, and heightened greenhouse gas emissions (Guo and Wang, 2021). Specifically, excessive nitrogen application led to substantial losses via ammonia volatilization, nitrate leaching, and denitrification processes. Consequently, this excessive application translated into higher fertilizer costs, reduced crop yields, escalated production expenses, and severe environmental pollution (Yang et al., 2024). Conversely, as nitrogen and phosphorus fertilizers became increasingly prevalent in farmland and crop yields soared, the demand for soil potassium by crops also saw a marked increase (Chen et al., 2021). Simultaneously, the persistent neglect of potassium fertilizer application resulted in a chronic deficiency of soil potassium (Das et al., 2021). In numerous regions, the extent of soil potassium deficiency was progressively expanding, the severity of the deficiency was intensifying, and the benefits of crop potassium application were becoming increasingly pronounced.

The direction of future fertilizer research ought to concentrate on enhancing fertilizer efficiency and utilization, rather than merely escalating fertilization levels (Martínez-Dalmau et al., 2021). The rise of controlled-release fertilizers stand as a pivotal trend in the evolution of fertilizer technology, promising to bolster fertilizer utilization efficiency, mitigate environmental pollution stemming from fertilization, and facilitate single-application convenience (Vejan et al., 2021). These innovative fertilizers operated by employing polymer coatings and other mechanisms to achieve nutrient release according to a predefined pattern, thereby aligning with the nutrient absorption patterns of crops.

Controlled-release fertilizers were widely utilized in cereal crops, including corn, wheat, and rice, highlighting their benefits in conserving both fertilizer and labor (Fan et al., 2021; Govil et al., 2024). These fertilizers, specifically those with controlled-release nitrogen, fostered enhanced root development, augmented wheat’s capacity to absorb soil nutrients, mitigated nitrogen losses, and bolstered overall fertilizer use efficiency (Liu et al., 2023; Xiang et al., 2025). When compared to conventional urea, controlled-release nitrogen fertilizers elevated the SPAD values and photosynthesis rates of wheat leaves, ultimately leading to increased crop yields (Ma et al., 2021). Additionally, controlled-release potassium fertilizers markedly boosted the levels of available potassium in the soil. In accordance with wheat’s growth and developmental nutrient requirements, potassium was steadily released from the soil, ensuring a continuous supply to sustain normal wheat growth (Li et al., 2021). The synergistic interaction between nitrogen and potassium further optimized the utilization efficiency of these nutrients in wheat and facilitated enhanced uptake of nitrogen, phosphorus, and potassium by both wheat grains and straw (Badawy et al., 2021).

Controlled-release fertilizers were at that time a prominent area of interest in international fertilizer research and were seen as a crucial path for future development in the fertilizer industry (Channab et al., 2023). Although the effects of controlled-release N and K fertilizers on plant had been widely studied, long-term field studies focusing on their combined application and the underlying physiological and biochemical mechanisms in wheat systems remain limited. Consequently, the purpose of this study was to examine the influence of combining controlled-release nitrogen fertilizer with controlled-release potassium fertilizer on various aspects: (i) photosynthetic properties, (ii) nitrogen and potassium utilization efficiency, (iii) soil nutrient movement, (iv) wheat yield, and (v) economic advantage. This study sought to explain the physiological and biochemical rationale behind the combined use of controlled-release nitrogen and controlled-release potassium to enhance wheat yield, thereby developing effective fertilization strategies for achieving high-yield wheat cultivation.

## Materials and methods

### Experimental site and material

**Figure 1:**
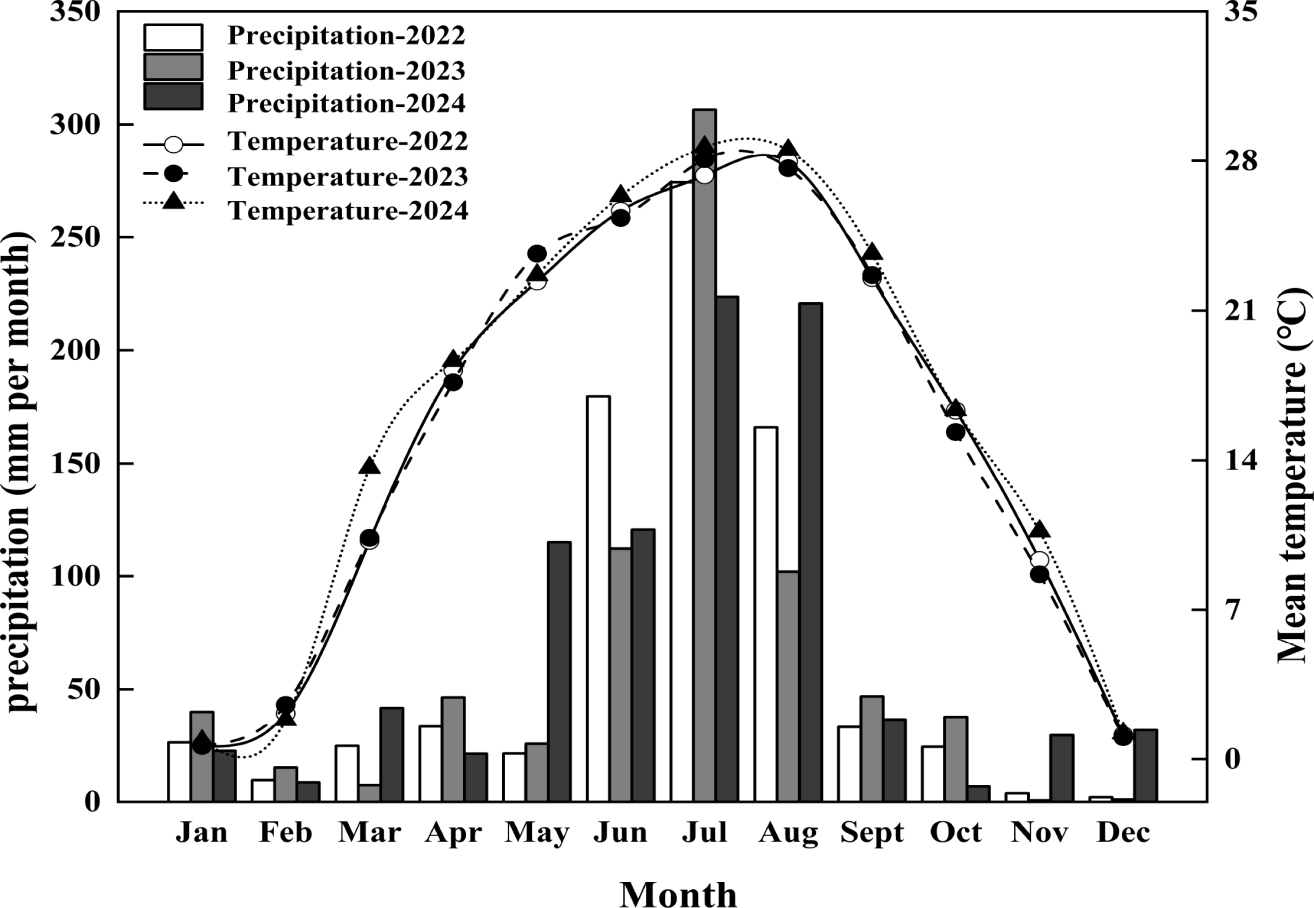
Weather data.

The experimental endeavor was carried out between the years 2022 and 2024 in Guxian Village, located in Zhongce County, Jining City, Shandong Province, China (N 35°69′22″; E 117°27′26″). This locale featured a warm temperate monsoon climate characterized by seasonal precipitation concentration. Precipitation was predominantly observed during the months of July and August, while the annual mean temperature was depicted in **Figure 1**. According to the USDA classification system (Soil Survey Staff, 1999), the soil type at the experimental site was identified as Typic Hapludalf. Prior to wheat cultivation, soil samples from the 0–20 cm depth were gathered using the 5-point sampling technique within the experimental plot to ascertain the fundamental soil characteristics, as summarized in **Table 1**. The experimental area had been previously utilized for food crop cultivation, maintaining uniform soil conditions.

**Table 1:** Part properties of tested soil before wheat planting.

Year	pH value	Organic matter	Total N	NO_3_^−^-N	NH_4_^+^-N	Available P	Available K
	(2.5:1)	(g kg^-1^)	(g kg^-1^)	(mg kg^-1^)	(mg kg^-1^)	(mg kg^-1^)	(mg kg^-1^)
2022	7.56	12.0	1.04	58.74	35.21	38.28	120.30
2023	7.62	12.0	1.05	62.32	36.58	38.31	123.74

The wheat variety chosen for planting was “Jimai 22”. The fertilizers under investigation encompassed controlled-release fertilizers and traditional fertilizers. The controlled-release fertilizer consisted of polymer-coated urea (CRU, containing 41% N, with nutrient release spanning nearly four months in 25 C static water) and polymer-coated potassium chloride (CRK, containing 55% K_2_O, with nutrient release spanning nearly four months in 25 C static water), both supplied by Kingenta Ecological Engineering Group Co., Ltd., China. Additionally, the traditional fertilizers included urea (containing 46% N) and calcium superphosphate (containing 14% P_2_O_5)_.

### Experimental design

The experiment employed a split-plot design, assigning nitrogen fertilizer type as the main plot factor and potassium fertilizer rate as the subplot factor, both arranged at random with three repetitions. The main plots featured two nitrogen fertilizer types: controlled-release urea (CRU) and conventional urea (Urea). Within the subplots, potassium fertilizer rates (CRK) were allocated at 50kg ha^-1^ (LCRK), 75kg ha^-1^ (MCRK), and 100kg ha^-1^ (HCRK). A control plot was also established, devoid of nitrogen and potassium applications. Each subplot measured 25 m^2^ (5m wide and 5m long). Urea fertilizers were applied in two installments: 40% pre-planting and 60% as topdressing at the elongation stage, while all other fertilizers were applied once prior to planting. The application rates amounted to 225kg ha^-1^ N and 150kg ha^-1^ P_2_O_5_.

To mitigate the impact of water and fertilizer infiltration across subplots, each subplot was delineated by a cement board with a thickness of 80mm and buried to a depth of 1 meter. Each subplot hosted 20 wheat rows, alternating between wide and narrow rows, with widths of 25cm and 15cm, respectively. Fertilization was conducted in furrows within the central strip of the wide rows. Wheat was sown at a rate of 120kg ha^-1^, with seeds buried 3cm deep. Fertilizers were buried in trenches within the wide rows, spanning 10–15 cm in width. The ratio of wheat rows to fertilizer rows was maintained at 2:1. Consistent with local agricultural practices, all other agricultural management measures were uniformly implemented.

### N and K release rates of CRU and CRK

The nutrient extraction technique, which involved utilizing static water within an indoor incubator maintained at a constant temperature of 25 C, was established in accordance with the industry standard titled “Controlled Release Fertilizer” (HG/T 4215-2011). For the nutrient release process of bags buried in field soil, a plastic sealing machine was employed to compress a nylon mesh, featuring a pore size of 1mm, into a bag measuring 12cm in length and 10cm in width. Subsequently, 10.0 grams of the coated CRU or CRK sample was weighed, placed into the prepared mesh bag, sealed using the plastic sealing machine, and buried 15cm deep in the field soil. Regarding the collection of fertilizer bags, during the wheat-growing season, they were retrieved at intervals of 10 days, 20 days, 30 days, and so forth, up to 240 days after burial. On each occasion, three bags were collected and transported back to the laboratory. These bags were then meticulously rinsed with pure water, dried in a 60 C oven for 48 hours, cooled in a dryer, and subjected to analysis. The total nitrogen content was determined using the semi-micro Kjeldahl method, while the total potassium content was measured using the flame method. The nutrient release rate at each time point was calculated by subtracting the total nutrient content of the retrieved fertilizer bag from the initial nutrient content at the time of burial, and then dividing this difference by the initial nutrient content of the buried bag.

### Soil sampling and measurement

Wheat crops were cultivated on October 14th, 2022, and October 17th, 2023. Throughout specific growth stages of the wheat, namely, the tillering stage (38 days post-sowing), the elongation stage (179 days post-sowing), the heading stage (213 days post-sowing), and the maturity stage (235 days post-sowing). The soil samples were gathered from the 0–20 cm soil layer during the second cultivation season. In each plot, a five-point sampling technique was employed to collect and combine the soil samples. Subsequent to natural air drying, the soil samples underwent grinding and sieving processes, utilizing sieves with mesh sizes of 2mm and 0.2mm, to prepare them for analysis and chemical testing. Using 0.01 M CaCl_2_, the samples were extracted and filtered, and the soil inorganic nitrogen content (NO_3_^--^N and NH_4_^+^-N) was analyzed with an AA3-A001-02E automatic analyzer. Additionally, the samples were treated with 1 mol/L NH_4_OAc, and the soil’s available potassium content was determined through the flame photometry method, as outlined by Zheng et al. (2016).

### Plant sampling and measurement

After 38 days of wheat planting, distinctive plants within each experimental plot were designated as fixed observation points for monitoring wheat tillering. Subsequently, the number of wheat tillers was meticulously observed and documented every 14 days, continuing until the waxing stage. Upon harvest, representative wheat samples measuring 1 m^2^ were gathered. These samples were air-dried and threshed post-seeding, with the grains and straw subsequently separated to determine the wheat yield. Additionally, five representative plants were collected at harvest time, categorized into straw and grain samples, and dried at 80 C until they attained a consistent weight. These samples were then finely ground and sieved, enabling the calculation of biomass and the measurement of total nitrogen and potassium content. Ultimately, the methodologies for computing nitrogen recovery efficiency (NRE), nitrogen agronomic efficiency (NAE), potassium recovery efficiency (KRE), and potassium agronomic efficiency (KAE) were detailed by Yang et al. in 2016.

1. NRE (%)=(cumulative plant N uptake from N treatment- cumulative plant N uptake from the Control treatment)/the amount of N fertilizer applied×100%;
2. NAE (kg N kg^-1^)=(the yield in the N treatment-the yield in the Control treatment)/the amount of N fertilizer applied;
3. KRE (%)=(cumulative plant K uptake from K treatment- cumulative plant K uptake from the Control treatment)/the amount of K fertilizer applied×100%;
4. KAE (kg K_2_O kg^-1^)=(the yield in the K treatment-the yield in the Control treatment)/the amount of K fertilizer applied;
5. Net profit ($ ha^-1^ year^-1^)=the yield×wheat price-fertilizer costs-other costs-labor costs.

The mean prices of fertilizers and other non-labor expenses in China (US dollars per metric ton): polymer coated urea $348.4, polymer coated potassium chloride $485.6, calcium superphosphate $149.3, urea $238.8, wheat $403.8. Other costs included machinery, plastic film, irrigation, pesticides, insecticides, seeds, and other materials and services. Mean cost of labor in China: $12.80 for one employee/day/ha.

### Photosynthetic detection of wheat

At the elongation stage of wheat, which occurred 179 days after sowing, measurements were taken of chlorophyll content, photosynthesis, and chlorophyll fluorescence parameters. Specifically, two rows of plants from each experimental center were randomly selected for these assessments. The SPAD value was determined using a chlorophyll meter, model SPAD-502, manufactured by Minolta in Tokyo, Japan. Additionally, the net photosynthetic rate (*P*_n_), stomatal conductance (*G*_s_), intercellular carbon dioxide concentration (*C_i_*) and transpiration rate (*T*_r_) were all measured utilizing the Li-6400 portable photosynthetic apparatus, produced by LI-COR in Lincoln, Nebraska, USA. Furthermore, the primary light energy conversion efficiency (*F*_v_/*F*_m_), non-photochemical quenching coefficient (*q*_N_), photochemical quenching coefficient (*q*_P_), and effective quantum yield of PSII photochemistry (*Φ*PSII) were all evaluated using the FMS2 portable fluorescence system, a product of Hansatech Instruments based in Kings Lynn, Norfolk, UK.

### Statistical analyses

Data were preprocessed using Microsoft Excel 2010 and subjected to Tukey and Duncan multiple comparisons, as well as two-factor analysis of variance with split-plot design, using SPSS 19.0 statistical software. All data presented in this article are the mean values from three replicates, and figures were generated using SigmaPlot software (version 12, MMIV, Systat Software Inc., San Jose, CA, USA).

## Results

### Nutrient release characteristics of CRU and CRK in 25 C static water and field soil

**Figure 2:**
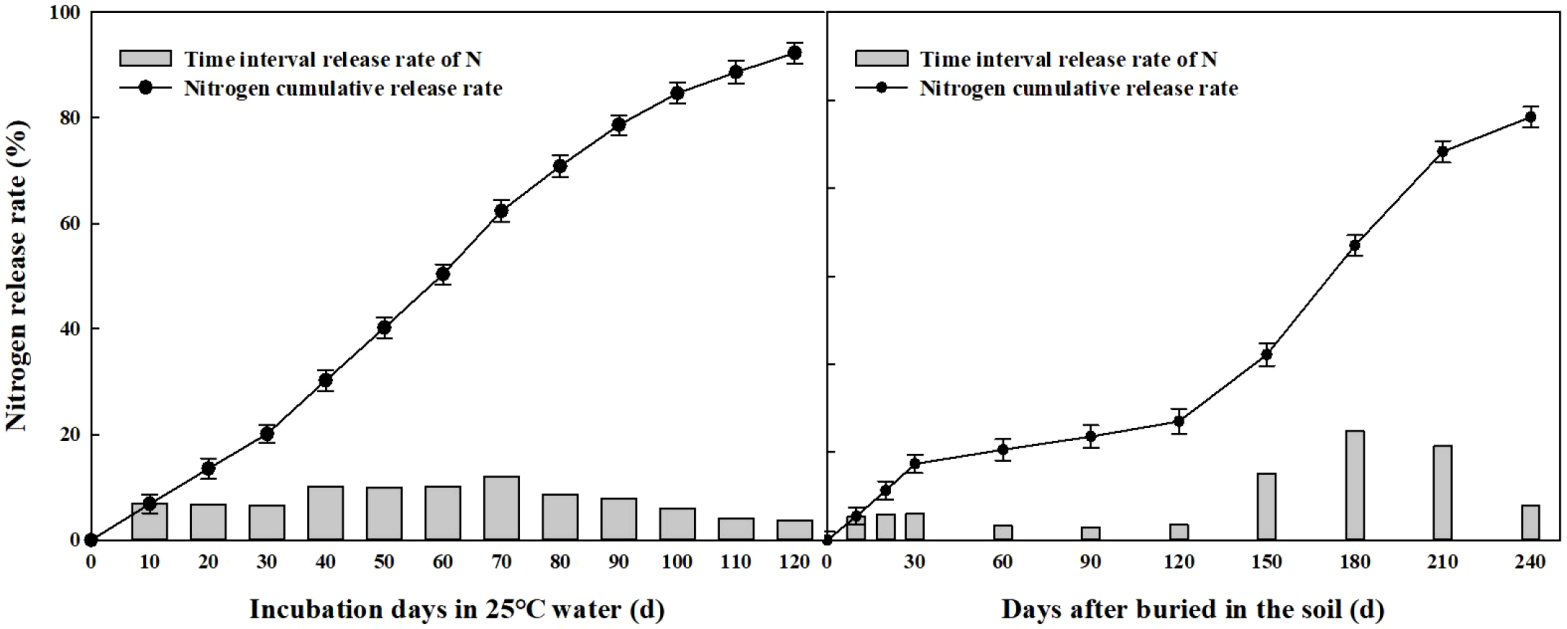
Nitrogen release rate of CRU in water and field condition.

**Figure 3:**
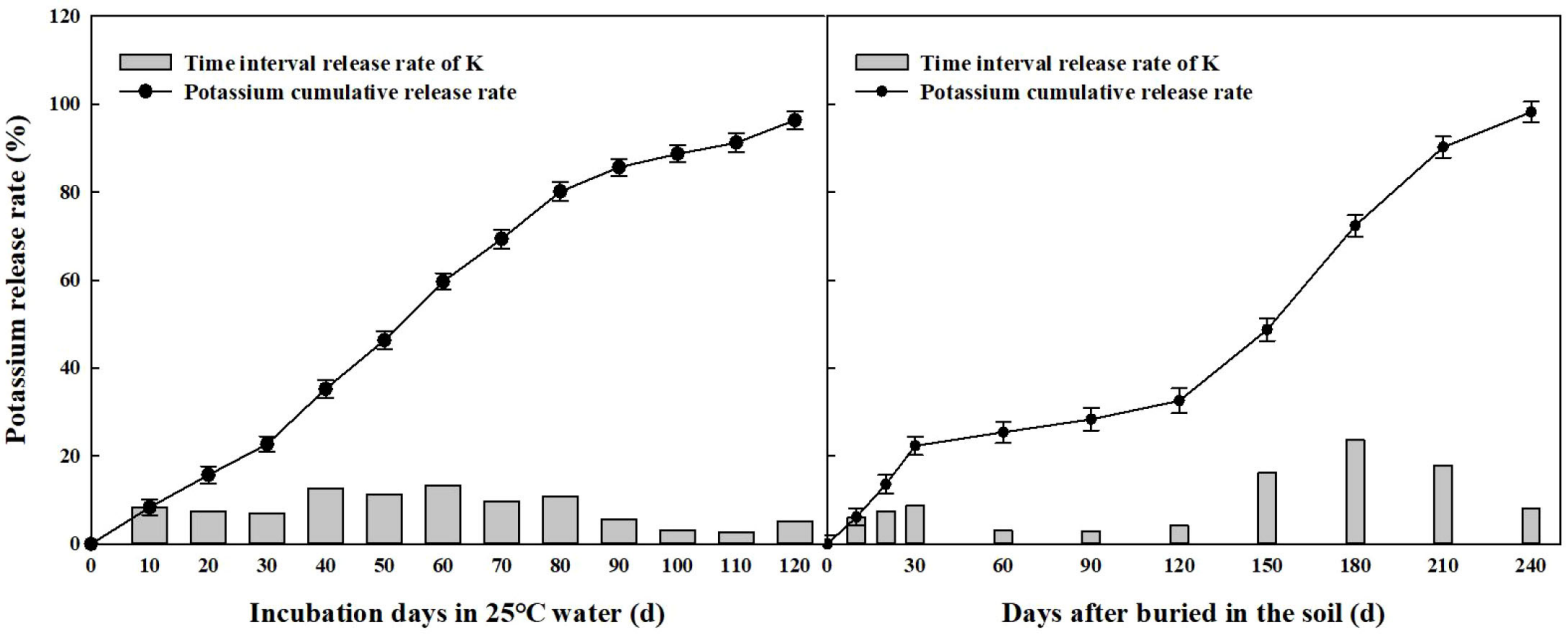
Potassium release rate of CRK in water and field condition.

During static water extraction conducted at 25 C, the nitrogen release profiles of CRU exhibited an “S” shaped pattern (**Figure 2**). Initially, nitrogen release was gradual over the first 30 days, peaking between days 40 and 80, and then gradually tapering off between days 90 and 120. Meanwhile, the potassium release profiles of CRK mirrored those of CRU but occurred at a quicker pace (**Figure 3**). After 120 days under these conditions, the nitrogen release of CRU and the potassium release curves of CRK amounted to 92.3% and 96.3%, respectively. The nitrogen release of CRU was 4.02% higher than the potassium release of CRK at this 120 day mark.

The soil temperature in the 0–15 cm layer during the wheat season in this experiment averaged 10.7 C, considerably cooler than the 25 C of the static water extraction setup (depicted in **Figure 1**). Consequently, the nutrient release dynamics of CRU and CRK in the soil during the wheat season unfolded in three distinct phases (**Figures 2**, **3**). Prior to wheat entering its overwintering stage (within the first 30 days), nutrients were swiftly released, followed by a “lag period” spanning from day 30 to day 120. During this period, wheat was in its overwintering stage, with an average soil temperature dipping below 6 C. Subsequently, from day 120 to day 210, as soil temperatures climbed, the controlled-release fertilizers accelerated their nutrient release until reaching peak levels. Between the two types, CRK exhibited the fastest nutrient release compared to CRU. By day 240, the cumulative nutrient release rates for CRU and CRK had reached 96.2% and 98.2%, respectively.

### Soil inorganic nitrogen content

**Figure 4:**
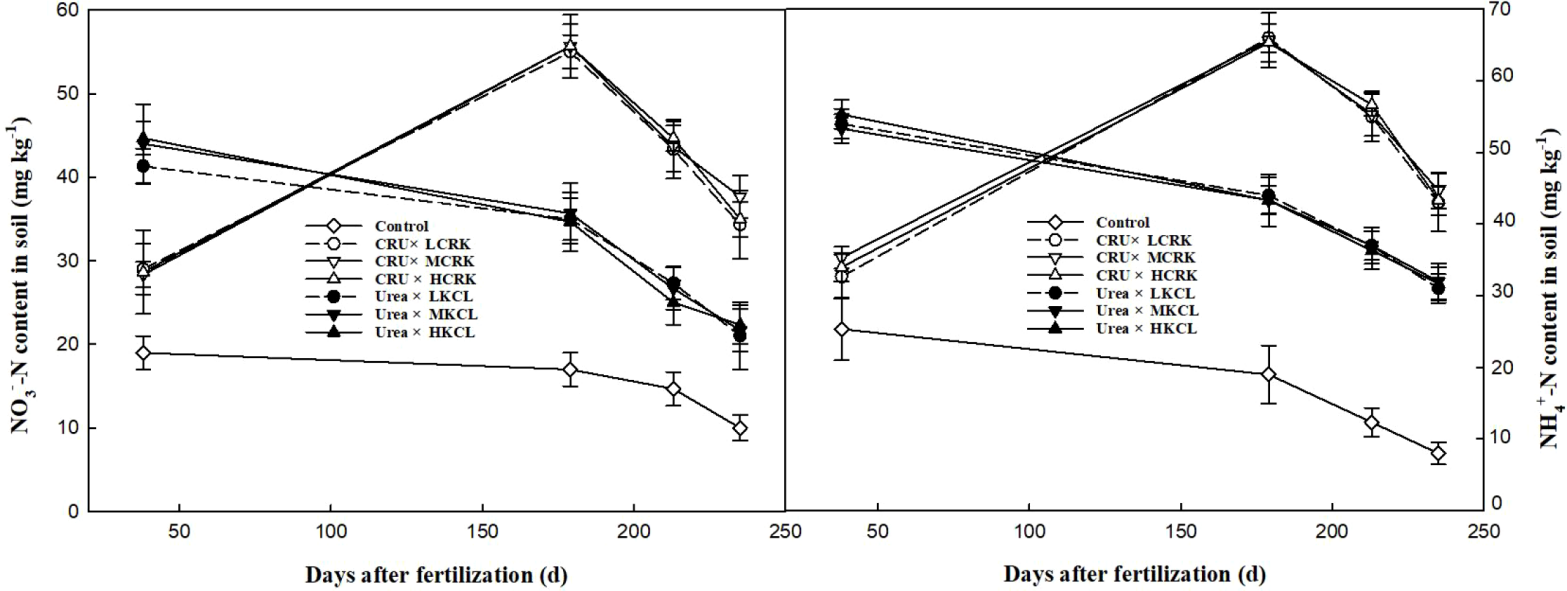
Changes of NO_3_^--^N and NH_4_^+^-N contents.

The levels of NO_3_^--^N in the soil of each nitrogen application treatment rose when compared to the non-nitrogen application treatment (Control). **Figure 4** shows that the application of controlled-release nitrogen fertilizer led to a substantial increase in nitrate nitrogen compared to the conventional urea treatment, highlighting that varying fertilization quantities influenced the nitrogen residuals in the soil across all fertilization methods. Specifically, in the 0–20 cm soil layer, wheat tillering stage treated with ordinary urea exhibited a 42.5-57.7% higher NO_3_^--^N content than those treated with CRU. This was attributed to immediate fertility of the urea, resulting in a rapid surge in NO_3_^--^N levels upon soil application. However, NO_3_^--^N carried a negative charge, and the adsorption capacity of soil for it was limited, which heightened the risk of NO_3_^--^N leaching and subsequently led to nitrogen losses and environmental contamination. With the growth of wheat, the advantage of slow release of CRU was highlighted. The NO_3_^--^N content of the CRU treatment was higher than that of urea, and the CRU×MCRK and CRU×HCRK treatment reached the maximum value of 55.6 mg kg^-1^ in the elongation stage. In the wheat harvest period, the NO_3_^--^N of CRU treatments were 63.5-79.4% higher than urea. As wheat grew, the NO_3_^--^N content in this soil layer decreased, indicating that controlled-release fertilizer potentially retained soil nitrogen and mitigated the risk of NO_3_^--^N leaching.

Similarly, **Figure 4** shows that the Control treatment resulted in a marked decrease in soil NH_4_^+^-N content. During the wheat seedling and heading stages, soil treated with ordinary urea had higher ammonium nitrogen levels compared to various controlled-release urea treatments, potentially accelerating ammonium volatilization and leaching, thereby causing nitrogen losses. During the wheat seedling stage, nitrogen-fertilized soil had relatively high NH_4_^+^-N content, whereas NH_4_^+^-N levels remained relatively stable in soil during other growth stages. In the 0–20 cm soil layer, during the wheat greening period, each CRU treatment had a 48.5-52.3% higher soil NH_4_^+^-N content than the urea treatment. This was due to the peak release of CRU occurring around the wheat elongation period, which CRU×LCRK treatment reached the maximum value of 66.0 mg kg^-1^. Conversely, during the winter wheat tillering stage, urea-treated soil had a higher NH_4_^+^-N content than soil treated with CRU. However, in later wheat growth stages, there were no significant differences in soil NH_4_^+^-N content among CRU treatments.

### Soil potassium form

**Figure 5:**
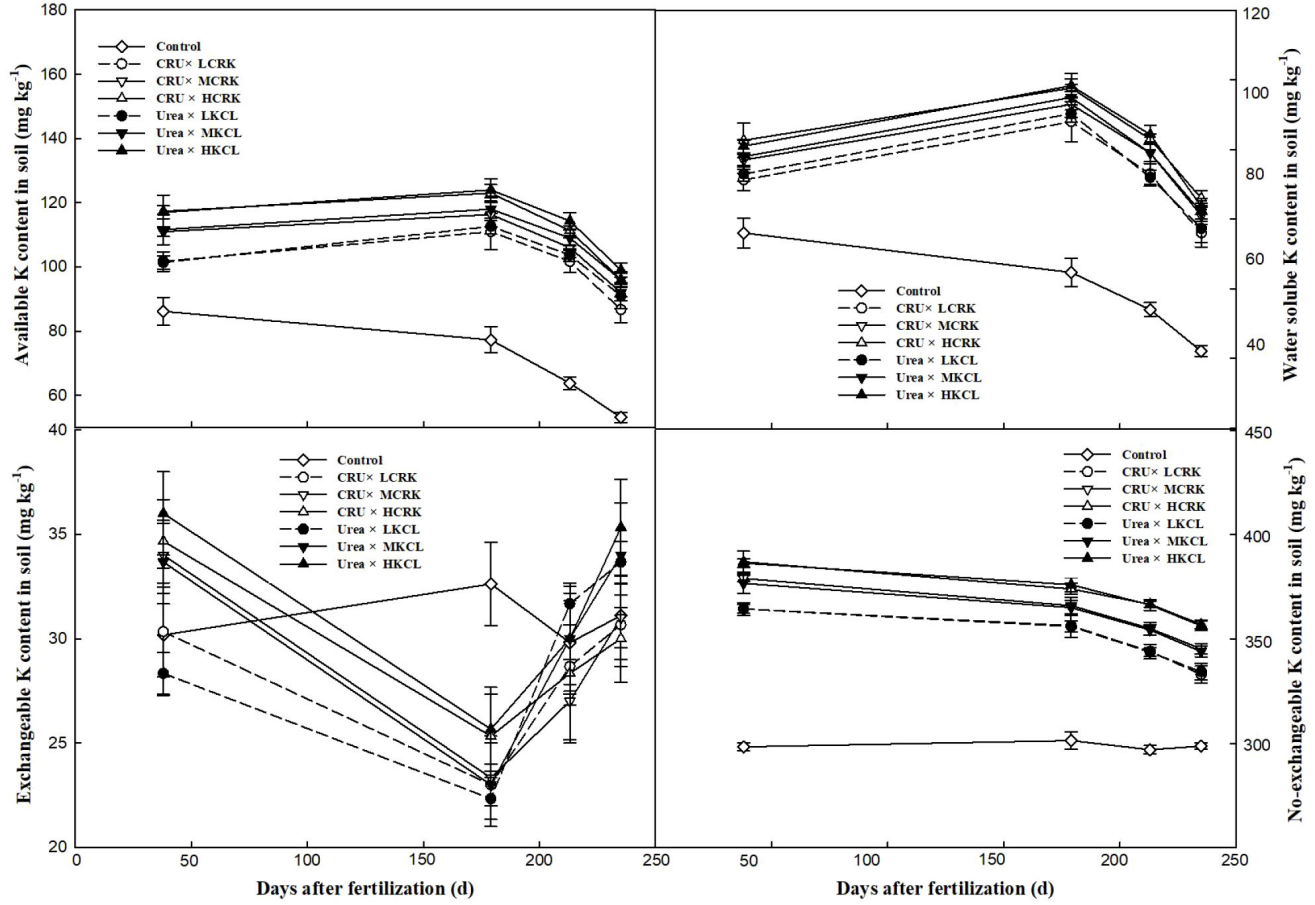
Changes in the soil K forms.

The **Figure 5** shows that the contents of soil available K, water soluble K, exchangeable K and nonexchangeable K were influenced by the quantity of CRK administered. Throughout various stages of development, the Control treatment exhibited the lowest concentrations. During the initial phase of wheat growth, alterations in the soil available K, water soluble K, and nonexchangeable K contents were relatively minor, attributed to limited the potassium absorption of wheat at this stage. Upon reaching the elongation stage, wheat transitioned into a period of rapid growth, resulting in a modest decline in the concentrations of various potassium components. Generally, the levels of soil available K, water soluble K, and nonexchangeable K in the soil progressively declined, whereas exchangeable K exhibited a fluctuating pattern. Irrespective of the nitrogen fertilizer type utilized, an increase in the CRK application rate led to an elevation in the soil available K, water soluble K, and exchangeable K contents, with significant variations observed among different potassium fertilizer treatments.

### Photosynthetic detection of wheat leaves

**Figure 6:**
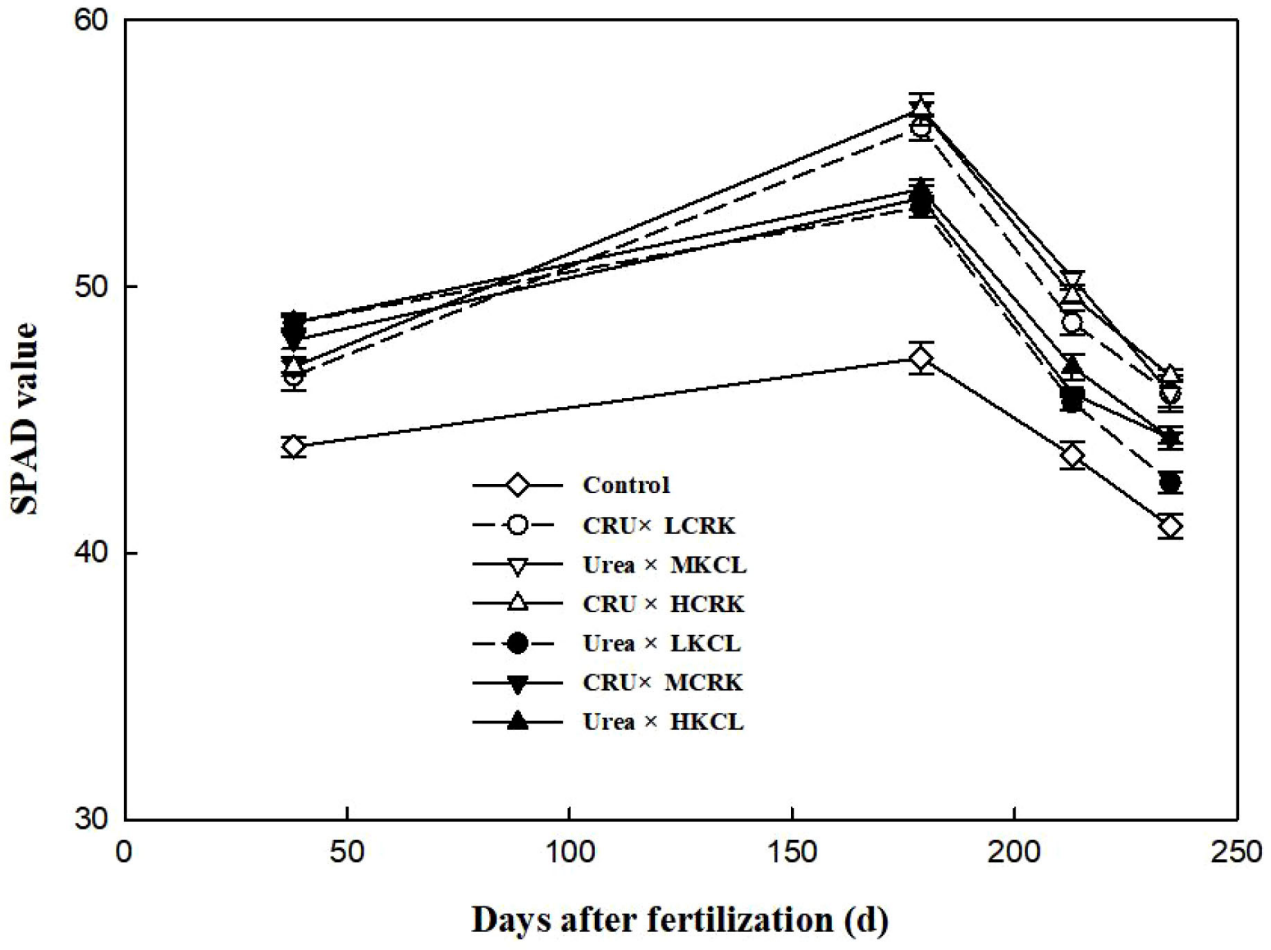
Changes of SPAD value.

The effects of various fertilization treatments on the SPAD values of wheat leaves were depicted in **Figure 6**. During the tillering stage of wheat, the SPAD values observed in the urea treatments surpassed those in the CRU treatments. Across the wheat’s entire growth period, the SPAD values initially rose and subsequently declined, peaking during the elongation stage, a time of vigorous nutritional growth. In the middle and later stages of wheat growth, the SPAD values associated with CRU treatments were 3.8-24.1% higher compared to those of urea treatments. Under three levels of CRK fertilizer application, the SPAD value of the LCRK treatment remained lower throughout the growth cycle, suggesting that inadequate potassium utilization had a discernible impact on the SPAD value. The CRU×HCRK treatment boasted the highest SPAD values during the middle and later stages of wheat growth, while the influence of nitrogen fertilizer on the SPAD value was more pronounced in the later stages. When compared to conventional urea, the application of CRU exhibited greater advantages in enhancing SPAD values.

In the past two years, the control treatment exhibited the lowest net photosynthetic rate (*P*_n_), stomatal conductance (*G*_s_) and transpiration rate (*T*_r_), while the intercellular carbon dioxide concentration (*C*_i_) was the highest, as indicated in **Table 2**. No significant interaction effect between nitrogen (N) and potassium (K) was observed on the photosynthesis indicators. Among the treatments, the CRU×MCRK and CRU×HCRK treatments demonstrated the most favorable performance in terms of wheat leaf photosynthesis. In comparison to the urea treatments, the CRU treatments showed a significant increase in photosynthetic factors. As the potassium fertilizer dosage increased, there was no notable difference in *P*_n_ among treatments using the same type of nitrogen fertilizer. Furthermore, no significant difference was found in *G*_s_, *T*_r_ and *C*_i_ between the MCRK and HCRK treatments, but these values were higher than those of the LCRK treatment. Both nitrogen fertilizer types and potassium fertilizer rates influenced the photosynthesis indicators, yet their interaction effects did not yield a significant difference.

**Table 2:** Parameters of photosynthesis chlorophyll and fluorescence of wheat leaf at jointing stage, 2024.

Treatment	*P*_n_	*G*_s_	*T*_r_	*C*_i_	*Φ*PSII	*F*_v_/*F*_m_	*q*_P_	*q*_N_
	(umol m^-2^ s^-1^)	(umol mol^-1^)	(umol m^-2^s^-1^)	(umol m^-2^ s^-1^)				
Nitrogen fertilizer type								
CRU	16.49 a	0.58 a	10.57 a	213.78 b	0.58 a	0.78 a	0.88 a	1.22 b
Urea	15.69 b	0.54 b	9.64 b	229.33 a	0.54 b	0.75 b	0.86 b	1.26 a
Potassium fertilizer rate (kg ha^-1^)								
50	15.93 b	0.54 b	9.80 b	224.17 a	0.56 a	0.76 b	0.86 b	1.26 a
75	16.22 a	0.57 a	10.30 a	219.50 c	0.57 a	0.77 a	0.87 a	1.23 b
100	16.12 a	0.56 a	10.22 a	221.00 b	0.56 a	0.77 a	0.87 a	1.24 b
Nitrogen fertilizer type×Potassium fertilizer rate interaction								
Control	11.73 c	0.46 e	8.27 e	251.00 a	0.51 c	0.72 e	0.81 e	1.34 a
CRU×LCRK	16.30 a	0.56 bc	10.20 b	217.00 d	0.58 a	0.78 ab	0.87 bc	1.23 d
CRU×MCRK	16.63 a	0.59 a	10.80 a	211.33 e	0.59 a	0.79 a	0.89 a	1.21 e
CRU×HCRK	16.53 a	0.58 ab	10.70 a	213.00 e	0.59 a	0.78 ab	0.88 ab	1.22 de
Urea×LCRK	15.57 b	0.52 d	9.40 d	231.33 b	0.54 b	0.74 d	0.85 d	1.28 b
Urea×MCRK	15.80 b	0.55 c	9.80 c	227.67 d	0.55 b	0.76 c	0.86 cd	1.25 c
Urea×HCRK	15.70 b	0.55 c	9.73 c	229.00 bc	0.54 b	0.75 cd	0.86 cd	1.25 c
Source of variance (*P* value)								
Nitrogen fertilizer type	<0.0001	<0.0001	<0.0001	<0.0001	<0.0001	<0.0001	<0.0001	<0.0001
Potassium fertilizer rate	<0.0001	<0.0001	<0.0001	<0.0001	0.207	0.003	0.005	0.001
Nitrogen fertilizer type×Potassium fertilizer rate interaction	0.493	0.723	0.187	0.31	0.638	0.821	0.346	0.397

Means followed by different lowercase letters in the same column were significantly different based on analyses with ANOVAs followed by Duncan tests (*P* < 0.05).

Control, no nitrogen and potassium fertilizer; CRU, polymer-coated urea; CRK, polymer-coated potassium chloride; *P*_n_, photosynthetic parameters including net photosynthetic rate; *G*_s_, stomatal conductance; *C*_i_, intercellular carbon dioxide concentration; *T*_r_, transpiration rate; *Φ*PSII, the effective quantum yield of PSII photochemistry; *F*_v_/*F*_m_, the primary light energy conversion efficiency; *q*_P_, photochemical quenching coefficient; *q*_N_, non-photochemical quenching coefficient.

Over the course of these two years, the control treatment exhibited the lowest effective quantum yield of PSII photochemistry (*Φ*PSII), primary light energy conversion efficiency (*F*_v_/*F*_m_), photochemical quenching coefficient (*q*_P_), while the non-photochemical quenching coefficient (*q*_N_) was the highest, as detailed in **Table 2**. There was no significant N×K interaction effect on the fluorescence indicators. However, both the type of nitrogen fertilizer and the rate of potassium fertilizer had significant impacts on these indicators. When compared to the urea treatments, the CRU treatments showed a marked increase in fluorescence factors. Moreover, as the dosage of potassium fertilizer increased, no significant difference was observed among treatments using the same type of nitrogen fertilizer. The types of nitrogen fertilizer and CRK rates influenced the fluorescence indicators, but their interaction effects did not yield a significant difference. The CRU×MCRK treatments helped delay leaf senescence and ensured a steady supply of photosynthetic products necessary for wheat growth over these two years.

### Nutrient absorption and utilization efficiency

The application of nitrogen fertilizer led to a significant enhancement in wheat’s nitrogen absorption, as shown in **Table 3**. Specifically, the CRU treatments exhibited higher nitrogen uptake, nitrogen agronomy efficiency (NAE), and nitrogen recovery efficiency (NRE) compared to the urea treatments. No notable interaction was observed between nitrogen fertilizer type and potassium fertilizer rate in terms of nitrogen uptake, NAE, and NRE. Overall, the CRU×MCRK treatment demonstrated the highest nitrogen absorption and utilization efficiency.

**Table 3:** Nitrogen uptake, Nitrogen agronomy efficiency (NAE) and Nitrogen recovery efficiency (NRE) of wheat plant after harvested in 2024.

Treatment	Nitrogen uptake	NAE	NRE
	(kg ha-1)	(kg kg-1)	(%)
Nitrogen fertilizer type			
CRU	195.6 a	10.5 a	31.6 a
Urea	184.7 b	8.4 b	28.5 b
Potassium fertilizer rate (kg ha^-1^)			
50	189.7 a	9.4 a	29.9 a
75	190.0 a	9.6 a	30.1 a
100	189.7 a	9.4 a	30.0 a
Nitrogen fertilizer type×Potassium fertilizer rate interaction			
Control	117.7 d	-	-
CRU×LCRK	196.3 a	10.6 a	31.6 a
CRU×MCRK	195.0 a	10.5 a	31.6 a
CRU×HCRK	195.3 a	10.5 a	31.6 a
Urea×LCRK	183.0 c	8.2 c	28.3 c
Urea×MCRK	187.0 b	8.6 b	28.7 b
Urea×HCRK	184.0 b	8.4 bc	28.4 bc
Source of variance (*P* value)			
Nitrogen fertilizer type	<0.0001	<0.0001	<0.0001
Potassium fertilizer rate	0.417	0.331	0.22
Nitrogen fertilizer type×Potassium fertilizer rate interaction	0.095	0.145	0.133

Means followed by different lowercase letters in the same column were significantly different based on analyses with ANOVAs followed by Duncan tests (*P* < 0.05).

Control, no nitrogen and potassium fertilizer; CRU, polymer-coated urea; CRK, polymer-coated potassium chloride.

Similarly, the application of potassium fertilizer boosted the potassium absorption of wheat, as indicated in **Table 4**. The potassium absorption increased with the rise in CRK dosage. However, the trends for potassium agronomy efficiency (KAE) and potassium recovery efficiency (KRE) were inverse. No significant interaction between nitrogen (N) and potassium (K) was observed in relation to potassium uptake, KAE, and KRE. Overall, the CRU×LCRK treatment showed the highest nutrient absorption and utilization efficiency among all treatments.

**Table 4:** Potassium uptake, potassium agronomy efficiency (KAE) and potassium recovery efficiency (KRE) of wheat plant after harvested in 2024.

Treatment	Potassium uptake	KAE	KRE
	(kg ha-1)	(kg kg-1)	(%)
Nitrogen fertilizer type			
CRU	107.7 a	11.1 a	38.2 a
Urea	106.8 a	11.1 a	38.3 a
Potassium fertilizer rate (kg ha^-1^)			
50	94.8 c	12.4 a	39.5 a
75	108.3 b	11.5 b	38.6 b
100	118.5 a	9.5 c	36.6 c
Nitrogen fertilizer type×Potassium fertilizer rate interaction			
Control	78.3 d	-	-
CRU×LCRK	95.0 c	12.4 a	39.4 a
CRU×MCRK	109.0 b	11.4 b	38.5 b
CRU×HCRK	119.0 a	9.7 c	36.5 c
Urea×LCRK	94.7 c	12.5 a	39.6 a
Urea×MCRK	107.7 b	11.6 b	38.7 b
Urea×HCRK	118.0 a	9.3 d	36.6 c
Source of variance (*P* value)			
Nitrogen fertilizer type	0.219	1	0.162
Potassium fertilizer rate	<0.0001	<0.0001	<0.0001
Nitrogen fertilizer type×Potassium fertilizer rate interaction	0.834	0.028	0.913

Means followed by different lowercase letters in the same column were significantly different based on analyses with ANOVAs followed by Duncan tests (*P* < 0.05).

Control, no nitrogen and potassium fertilizer; CRU, polymer-coated urea; CRK, polymer-coated potassium chloride.

### Yield And net profit

The wheat yield and its components under various fertilization treatments have been summarized in **Table 5**. There was no significant difference in wheat yield between the 2022–2023 and 2023–2024 seasons. However, nitrogen application treatments boosted wheat yield compared to treatments without nitrogen application. Specifically, when compared to ordinary urea treatments, the CRU treatment led to a notable increase in wheat yield. In both 2022–2023 and 2023-2024, the wheat yield under CRU treatments was 2.2% higher, averagely, than that under urea treatments. Furthermore, the CRU application increased the number of spikes and grains per spike, but had no notable influence on the thousand-grain weight (TGW) of wheat. Nevertheless, there was no significant difference between the MCRK and HCRK application treatments, both of which showed a marked increase compared to the LCRK application treatments. In both years, no significant interaction effect between N×K was observed on wheat yield and its components.

**Table 5:** Wheat yield and yield component under different treatments during 2023 and 2024 growing seasons.

	2023					2024				
Treatment	Spikes	Grains	TGW	Yield	Yield increase	Spikes	Grains	TGW	Yield	Yield increase
	(m^−2^ )	(spike^−1^ )	(g)	(kg ha^−1^)	(%)	(m^−2^ )	(spike^−1^ )	(g)	(kg ha^−1^)	(%)
Nitrogen fertilizer type										
CRU	714.8 a	40.6 a	40.5 a	7062.2 a		736.9 a	39.4 a	40.5 a	7328.3 a	
Urea	689.0 b	39.6 b	40.4 a	6956.9 b		719.2 b	39.7 a	40.4 a	7181.1 b	
Potassium fertilizer rate (kg ha^-1^)										
50	694.8 b	39.7 b	40.4 a	6976.0 c		722.8 c	39.9 a	40.5 a	7181.2 b	
75	706.3 a	40.3 a	40.6 a	7034.0 a		732.0 a	38.7 a	40.5 a	7295.3 a	
100	704.5 a	40.2 b	40.4 a	7018.7 b		729.3 b	40.2 a	40.5 a	7287.7 a	
Nitrogen fertilizer type×Potassium fertilizer rate interaction										
Control	455.0 e	20.8 e	36.9 b	4871.3 e	-	468.3 e	20.3 b	37.2 b	5187.3 d	-
CRU×LCRK	707.3 b	40.2 b	40.4 a	7026.7 b	44%	733.0 b	40.2 a	40.4 a	7240.3 b	40%
CRU×MCRK	719.0 a	40.8 a	40.6 a	7086.3 a	45%	740.0 a	37.5 a	40.6 a	7371.0 a	42%
CRU×HCRK	718.0 a	40.7 a	40.5 a	7073.7 a	45%	737.7 a	40.6 a	40.5 a	7373.7 a	42%
Urea×LCRK	682.3 d	39.2 d	40.5 a	6925.3 d	42%	712.7 d	39.5 a	40.5 a	7122.0 c	37%
Urea×MCRK	693.7 c	39.8 c	40.5 a	6981.7 c	43%	724.0 c	39.9 a	40.4 a	7219.7 bc	39%
Urea×HCRK	691.0 c	39.7 c	40.3 a	6963.7 c	43%	721.0 c	39.8 a	40.4 a	7201.7 bc	39%
Source of variance (*P* value)										
Nitrogen fertilizer type	<0.0001	<0.0001	0.351	<0.0001		<0.0001	0.788	0.618	<0.0001	
Potassium fertilizer rate	<0.0001	<0.0001	0.316	<0.0001		<0.0001	0.533	0.978	0.005	
Nitrogen fertilizer type×Potassium fertilizer rate interaction	0.876	0.94	0.371	0.821		0.008	0.426	0.757	0.683	

Means followed by different lowercase letters in the same column were significantly different based on analyses with ANOVAs followed by Duncan tests (*P* < 0.05).

Control, no nitrogen and potassium fertilizer; CRU, polymer-coated urea; CRK, polymer-coated potassium chloride.

The average annual income, cost, and net profit for various treatments in 2023 and 2024 were computed, revealing that the Control treatment had the lowest values, as indicated in **Table 6**. The results of the economic analysis showed that, despite the higher fertilizer input cost of CRU treatments compared to ordinary urea treatments, the benefits associated with CRU application were greater. This was attributed to the higher yields obtained with CRU treatments, leading to increased total benefits. Additionally, CRU treatments resulted in savings on labor costs due to reduced fertilizer input requirements. The findings indicated that, in comparison to urea treatments, CRU treatments increased net profit by 2.0% in 2023 and 3.3% in 2024. Regardless of the type of nitrogen fertilizer used, the MCRK dosage provided the highest economic benefits compared to LCRK and HCRK treatments. Overall, the CRU×MCRK treatment offered the greatest economic advantages.

**Table 6:** Mean annual revenue, costs, and net profits from potassium treatments (2023 and 2024).

Treatment	Total revenue		Fertilizer costs	labor cost	Other costs	Net profit		Change vs Control (%)	
	2023	2024	($ ha^-1^ year-^1^)			2023	2024	2023	2024
Control	1967	2095	187	150	500	1130	1258	-	-
CRU×LCRK	2837	2924	416	150	500	1771	1857	57	48
CRU×MCRK	2861	2976	440	150	500	1772	1887	57	50
CRU×HCRK	2856	2977	461	150	500	1745	1866	54	48
Urea×LCRK	2796	2876	346	200	500	1750	1830	55	45
Urea×MCRK	2819	2915	369	200	500	1750	1846	55	47
Urea×HCRK	2812	2908	391	200	500	1721	1817	52	44

Mean prices of fertilizers and other non-labor expenses in China (US dollars per metric ton): polymer coated urea $348.4, polymer coated potassium chloride $485.6, calcium superphosphate $149.3, urea $238.8, wheat $403.8. Other costs included machinery, plastic film, irrigation, pesticides, insecticides, seeds, and other materials and services. Mean cost of labor in China: $12.80 for one employee/day/ha.

Control, no nitrogen and potassium fertilizer; CRU, polymer-coated urea; CRK, polymer-coated potassium chloride.

## Discussion

### Nutrient leaching

Previous studies have shown that the long-term application of ammonium nitrogen fertilizer or urea could easily lead to an imbalance in the absorption of anions and cations by soil roots, causing soil acidification (Klimczyk et al., 2021). When NO_3_^--^N and NH_4_^+^-N were the main nitrogen sources, the total absorption of cations exceeded that of anions, exacerbating acidification (Yang et al., 2022). In this study, conventional urea significantly reduced soil alkaline cation concentration compared to controlled-release urea (CRU), which might have been related to its rapid hydrolysis promoting nitrate leaching (Cui et al., 2020). NH_4_^+^-N was adsorbed by soil and released H^+^, increasing acidity and causing other cations to be displaced into solution or deep soil. CRU potentially alleviated this process through slow-release nitrogen. The nutrient release of CRU was regulated by soil temperature and humidity. In the experiment, the average soil temperature before overwintering of wheat was 10.25 C (**Figure 1**), at which point the nutrients released by CRU could meet the initial nitrogen requirements of wheat. When the soil temperature dropped below 0.5 C in winter, the release almost stopped. After greening, as the temperature rose, nutrient release accelerated and reached its peak at the heading stage, accurately matching the nitrogen efficiency requirements of wheat at its highest stage. In summary, the release characteristics of CRU in the field were highly synchronized with the nitrogen requirements of wheat at different growth stages (Fan et al., 2022). In addition, the content of exchangeable potassium in CRU treatment was lower than that in common urea treatment. This indicated that the stable nitrogen supply in CRU treatment may promote the growth of roots, which might also enhance the absorption of nutrients such as potassium. This explains the reason for the increase in nitrogen and potassium absorption efficiency shown in **Tables 3** ,**4**, which might made a potential reduction in leaching.

### Nutrient use efficiency

With the advancement of agricultural production and the increase in fertilizer dependence, improving nutrient utilization efficiency had become crucial (Salim and Raza, 2020). Previous experiments had shown that controlled-release urea (CRU) improved the yield and nutrient efficiency of wheat and corn (Zhu et al., 2020; Zhang et al., 2022; Hou et al., 2024). Specifically, compared to traditional urea, CRU had increased nitrogen utilization efficiency by 28.5%, and even with a 33% reduction in nitrogen, wheat yield had increased by 6.5% (Sapkota and Takele, 2023). Our experiment found that under the same nitrogen fertilizer application rate, CRU moderately improved nitrogen absorption, agronomic efficiency (NAE), and recovery efficiency (NRE), thereby increasing wheat yield. The utilization efficiency of potassium was not affected by the type of nitrogen fertilizer. Potassium absorption increased with the increase of application rate, but agronomic (KAE) and recovery efficiency (KRE) decreased. CRU promoted nitrogen absorption and aboveground biomass, which were key drivers of wheat yield. The three-stage release of CRU and CRK dynamically and accurately matches the nutrient requirements of wheat (**Figures 2**, **3**). Rapid release 30 days before overwintering to meet seedling growth, slow release at low temperatures during the overwintering period (30–120 days) to avoid loss, and accelerated release with temperature during the greening to jointing period (120–210 days), in line with the key fertilizer demand peak for panicle differentiation. The final cumulative release rate exceeds 96%, significantly reducing nutrient loss and improving utilization efficiency. In the testing process, the CRU with medium controlled-release potassium (MCRK) combination performed the best, proving to be the most suitable for wheat growth.

### Multidimensional correlation analysis between yield and various indicators

**Figure 7:**
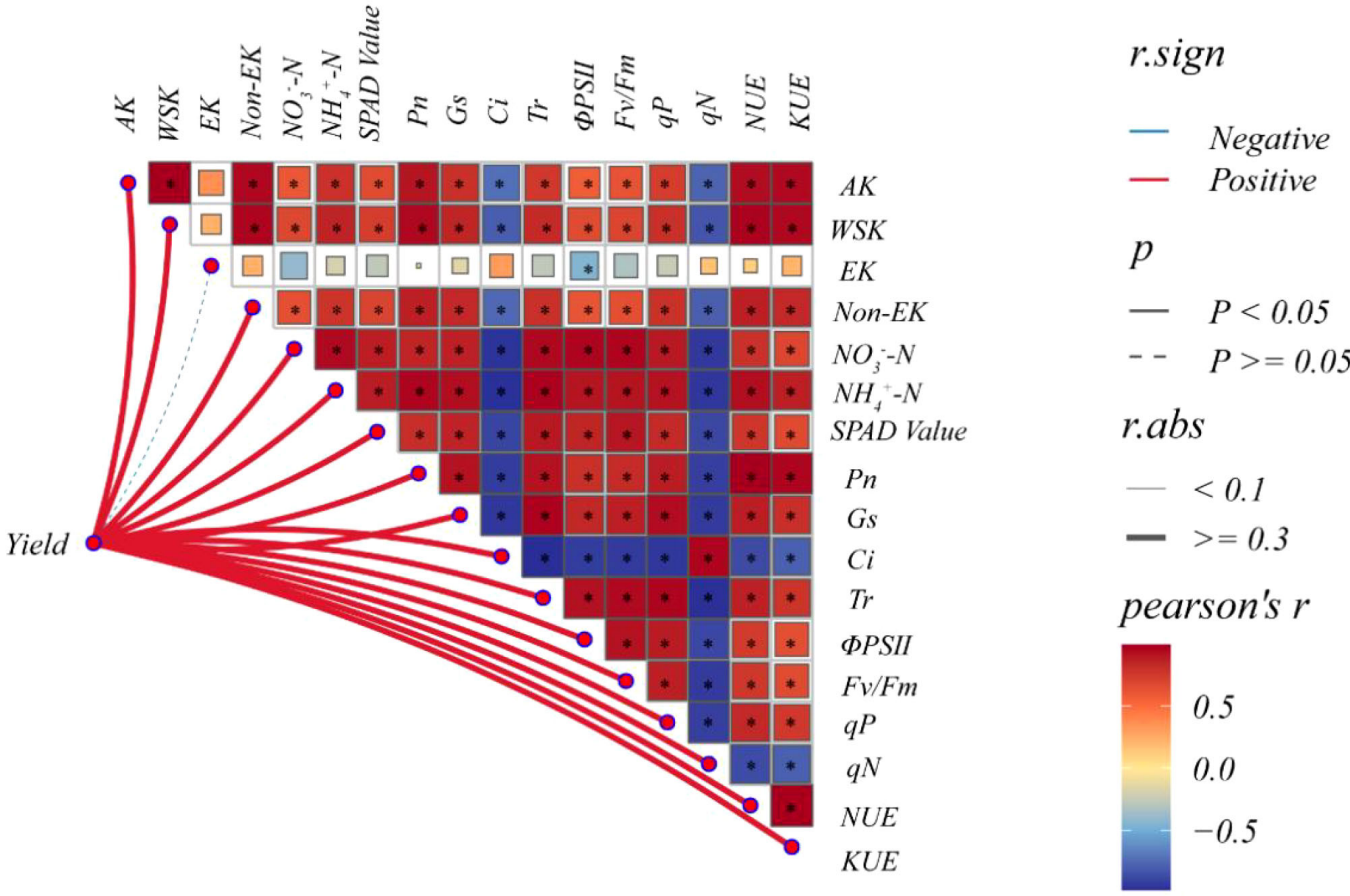
Multidimensional correlation analysis between yield and various indicators. AK, available K; WSK, water soluble K; EK, exchangeable K; Non-EK, Non-exchangeable K; *Indicates significant at the P0.05 level.

The yield per unit area of wheat is determined by the number of spikes, grains per spike, and TGW (Cossani and Sadras, 2021). This experiment reached similar conclusions. Higher potassium fertilizer rates gradually increased spike number per unit area. Controlled-release urea (CRU) outperformed ordinary urea in boosting spike number and thousand grain weight. However, high-rate controlled-release potassium (CRK) produced more spikes but lower TGW. Multidimensional correlation analysis (**Figure 7**) revealed key links. Soil available K, water-soluble K, non-exchangeable K, nitrate-N, ammonium-N, net photosynthetic rate (Pn), stomatal conductance (Gs), transpiration rate (Tr), SPAD value, and chlorophyll fluorescence parameters (ΦPSII, Fv/Fm, qP) all correlated positively with N and K use efficiency (P<0.05). Yield correlated positively with most soil nutrients, photosynthetic, and fluorescence indicators (P<0.05), except exchangeable K. The CRU with medium-rate CRK treatment balanced spike number, grains per spike, and TGW. It achieved super-high yield. Future research should focus on exploring how N×K interactions promote effective transportation of photosynthetic products and coordinate source sink relationships. And maintain cell osmotic potential and enzyme activity to support wheat’s absorption and utilization of nutrients. The purpose is to simultaneously improve photosynthetic efficiency and nitrogen fertilizer utilization efficiency.

## Conclusion

The nutrient release pattern of controlled-release fertilizers was primarily influenced by temperature, along with the coating material used. Consequently, when choosing the suitable type of controlled-release fertilizer, it was crucial to take into account both the growth cycle of crop and the soil temperature conditions in the planting area. In this study, a comparison was made between conventional urea application and a single application of controlled-release urea (CRU). Results indicated that the wheat yields in CRU treatments witnessed a modest average increase of 2.2% from 2022 to 2024 compared to ordinary urea treatments. The combination of CRU with a moderate level of potassium (CRU×MCRK) exhibited the most favorable effects. This treatment not only enhanced soil fertility and mitigated soil acidification but also improved nutrient use efficiency and promoted wheat growth compared to the standard urea treatment.

## Data Availability

The original contributions presented in the study are included in the article/supplementary material. Further inquiries can be directed to the corresponding authors.
